# Predicting early breast cancer recurrence from histopathological images in the Carolina Breast Cancer Study

**DOI:** 10.1038/s41523-023-00597-0

**Published:** 2023-11-11

**Authors:** Yifeng Shi, Linnea T. Olsson, Katherine A. Hoadley, Benjamin C. Calhoun, J. S. Marron, Joseph Geradts, Marc Niethammer, Melissa A. Troester

**Affiliations:** 1 Department of Computer Science, University of North Carolina at Chapel Hill, Chapel Hill, NC USA; 2 Department of Epidemiology, University of North Carolina at Chapel Hill, Chapel Hill, NC USA; 3 Lineberger Comprehensive Cancer Center, University of North Carolina at Chapel Hill, Chapel Hill, NC USA; 4 Department of Genetics, University of North Carolina at Chapel Hill, Chapel Hill, NC USA; 5 Department of Pathology and Laboratory Medicine, University of North Carolina at Chapel Hill, Chapel Hill, NC USA; 6 Department of Statistics and Operations Research, University of North Carolina at Chapel Hill, Chapel Hill, NC USA; 7 Department of Pathology, East Carolina University, Greenville, NC USA

**Keywords:** Epidemiology, Prognostic markers

## Abstract

Approaches for rapidly identifying patients at high risk of early breast cancer recurrence are needed. Image-based methods for prescreening hematoxylin and eosin (H&E) stained tumor slides could offer temporal and financial efficiency. We evaluated a data set of 704 1-mm tumor core H&E images (2–4 cores per case), corresponding to 202 participants (101 who recurred; 101 non-recurrent matched on age and follow-up time) from breast cancers diagnosed between 2008–2012 in the Carolina Breast Cancer Study. We leveraged deep learning to extract image information and trained a model to identify recurrence. Cross-validation accuracy for predicting recurrence was 62.4% [95% CI: 55.7, 69.1], similar to grade (65.8% [95% CI: 59.3, 72.3]) and ER status (66.3% [95% CI: 59.8, 72.8]). Interestingly, 70% (19/27) of early-recurrent low-intermediate grade tumors were identified by our image model. Relative to existing markers, image-based analyses provide complementary information for predicting early recurrence.

## Introduction

Early recurrence, herein defined as the return of a primary tumor within three years of diagnosis, is an important endpoint in clinical management of breast cancer^1^. Recurrences can often be successfully managed, but they are stressful, costly, and increase risk of mortality if not detected early^2–4^. Clinical risk stratification is currently based on several clinical characteristics, including hormone receptors, HER2 status, grade, stage, and age, and RNA-based methods are available to identify tumors with high risk of recurrence^5–8^. Clinical gene expression assays are not uniformly performed on all patients, and are often limited to specific subgroups of patients with low-stage and ER-positive disease. Genomic assays are also expensive^9^, so histopathology-based stratification is appealing. Currently, only combined histologic grade—a metric that classifies breast tumors according to tubule formation, nuclear pleomorphism, and mitotic frequency–is routinely collected from H&E images in the clinic. Grade evaluation is performed manually and is subject to interobserver variability. An objective, image-based method could be valuable for prescreening patients at higher risk of recurrence.

Recent work in computer vision has extensively explored using deep convolutional neural networks (CNNs) to extract global contextual information from a variety of image types. Early applications of CNNs primarily were focused on natural images (e.g., cars or birds)^10^, but more recently, methods have been extended to medical images, including radiographic and histopathologic images^11–13^. Machine learning methods in image classification have been shown to predict or diagnose invasive breast cancer incidence, using both histopathological and radiographic images^14–18^. However, few studies have evaluated breast cancer outcomes based on images, and most that have have been limited in sample size, range of tumor phenotypes, or patient diversity^19–21^. For example, there are several data sets (e.g., the Camelyon challenge in the Netherlands and IBM-curated BRIGHT) that have encouraged researchers to investigate benign and neoplastic breast tissue using machine learning methods; however, these are largely focused on diagnostic capacity rather than prognostic or predictive modeling. Campanella and colleagues used WSI from multiple cancers to develop a predictive model of invasive disease, but again, this work was focused on diagnostic rather than prognostic applications^16^. Many other previous studies have emphasized a priori hypotheses, such as associations with spatial arrangement of immune cells^22^ or emphasized overall or breast cancer-specific survival rather than recurrence. Furthermore, many data sets—even for diagnostics—do not include diverse populations of women with breast cancer. In the US, Black women have significantly higher recurrence rates and breast cancer mortality, but often have lower representation in clinical and observational research. We used data from a source that represented both Black and non-Black women in similar proportions, allowing us to investigate breast cancer recurrence in a diverse setting.

We sought to investigate whether we could use image information extracted with a CNN (VGG16^23^, a CNN pre-trained on ImageNet) together with support vector machines (SVM)^24^ to create image-based classes that were predictive of recurrence among breast cancer patients. We assessed reproducibility and inter- and intra-individual variance by comparing validation accuracy across and within patient specimens and compared results to existing, established biomarkers. The Carolina Breast Cancer Study (CBCS3) is a well-annotated image dataset for a diverse group of women (50% Black, 50% under age 50) who were followed for medical record-confirmed recurrence.

## Results

### Study population

Table 1 shows our study population and indicates that the matched dataset (*n* = 101) had a similar distribution relative to the full population represented on the tissue microarrays (TMAs, *n* = 1543). However, the subset of CBCS cases included on the TMA tended to include more large size tumors and higher-grade tumors relative to the entirety of CBCS. Relative to participants who experienced an early recurrence, age-matched participants without early recurrence were significantly more likely to be early stage (stage 1 52.5% vs 10.9% in early recurrences), grade 1 or 2 (58.4% vs 26.7% in early recurrences), and ER-positive (76.2% vs 43.6% in early recurrences).

**Table 1:** Demographic and clinical tumor characteristics for the full study population and the matched training sample, stratified by 3-year recurrence status.

		(*n* = 101)	Full Population (*n* = 1543)	Matched Sample (*n* = 101)	*p*-value*
		Recurrence	No Recurrence	No Recurrence	
Mean Number of Cores		3.62	3.44	3.35	0.27
Age		51.5 (10.7)	52.8 (11.2)	51.6 (10.6)	0.27
Race					1
	Non-Black	36 (34.7)	815 (52.8)	53 (52.5)	
	Black	66 (65.3)	728 (47.2)	48 (47.5)	
Grade					0.91
	1	6 (5.9)	343 (22.2)	22 (21.8)	
	2	21 (20.8)	592 (38.4)	37 (36.6)	
	3	74 (73.3)	608 (39.4)	42 (41.6)	
Stage					0.65
	1	11 (10.9)	737 (47.8)	53 (52.5)	
	2	52 (51.5)	646 (41.9)	38 (37.6)	
	3	38 (37.6)	160 (10.4)	10 (9.9)	
ER					0.74
	Positive	44 (43.6)	1199 (78.2)	77 (76.2)	
	Negative	57 (56.4)	335 (21.8)	24 (23.8)	
PR					0.89
	Positive	30 (29.7)	1051 (68.8)	70 (70.0)	
	Negative	71 (70.3)	477 (31.2)	30 (30.0)	
HER2					
	Positive	17 (16.8)	219 (14.3)	11 (10.9)	0.43
	Negative	84 (83.2)	1317 (85.7)	90 (89.1)	

*P-value for chi-squared test (categorical variables) or t-test (continuous variables) between non-recurrent participants in the full population and matched sample.

### Model prediction accuracy

First, we assessed the accuracy for detecting recurrence within three years of diagnosis in our balanced data set, displayed in Table 2. In cross-patient 10-fold cross validation we observed 62.4% accuracy and 63.4% sensitivity. However, using within-patient validation, accuracy was 70.3% (67.7% sensitivity). In both approaches, the sensitivity and specificity were well-balanced, with within-patients (72.9%, 95% CI: 64.2, 81.6) specificity slightly higher than sensitivity (67.7%, 95% CI: 58.6, 76.8) and cross-patients sensitivity (63.4%, 95% CI: 54.0, 72.8) slightly higher than specificity (61.4%, 95% CI: 51.9, 70.9). To contextualize these accuracy estimates, we also evaluated the accuracy of standard clinical markers. Using grade and ER status as predictors of recurrence resulted in accuracies of 65.8% and 66.3%, respectively, but grade had higher sensitivity (73.3%, 95% CI: 64.7, 81.9) while ER status had higher specificity (76.2%, 95% CI: 67.9, 84.5). In pre-screening tumors for genomic testing, sensitivity to detect aggressive tumors is higher priority.

**Table 2:** Recurrence prediction accuracy comparison between image-based classes and other tumor characteristics (ER, grade).

	Accuracy (95% CI)	Sensitivity (95% CI)	Specificity (95% CI)
Grade	65.8 (59.3, 72.3)	73.3 (64.7, 81.9)	58.4 (48.8, 68.0)
ER	66.3 (59.8, 72.8)	56.4 (46.7, 66.1)	76.2 (67.9, 84.5)
Within-Patients (Image Features)	70.3 (64.0, 76.6)	67.7 (58.6, 76.8)	72.9 (64.2, 81.6)
Cross-Patients (Image Features)	62.4 (55.7, 69.1)	63.4 (54.0, 72.8)	61.4 (51.9, 70.9)
**Among High Grade Tumors Only**			
ER	63.8 (55.0, 72.6)	68.9 (58.3, 79.5)	54.8 (39.6, 70.0)
Within-Patients (Image Features)	65.2 (56.5, 73.9)	66.7 (55.9, 77.5)	63 (48.2, 77.8)
Cross-Patients (Image Features)	53.4 (44.2, 62,6)	63.5 (52.5, 74.5)	35.7 (21.0, 50.4)
**Among Low/Intermediate Grade Tumors Only**			
ER	69.8 (60.2, 79.4)	22.2 (6.8, 37.6)	91.5 (84.4, 98.6)
Within-Patients (Image Features)	77.1 (68.3, 85.9)	70.4 (53.5, 87.3)	80.4 (70.4, 90.4)
Cross-Patients (Image Features)	61.6 (51.4, 71.8)	48.1 (29.6, 66.6)	67.8 (56.0, 79.6)

To investigate recurrence prediction accuracy among clinically low or high-risk tumors, we further stratified our accuracy assessment by grade (low/intermediate vs high) (Table 2). Accuracy was higher in the low/intermediate grade group compared to high grade for both the within-patients approach (77.1% vs 65.2% in low vs. high grade) and the cross-patients validation approach (61.6% vs. 53.4% in high-grade). The sensitivity was lower among low/intermediate-grade tumors, while specificity was lower for high-grade tumors. However, sensitivity of both image-based approaches in the low/intermediate group exceeded that for ER status (70.4% for within-patients, 48.1% for cross-patients vs. 22.2% for the ER status). A total of 19 low/intermediate group patients (70% of patients with low/intermediate grade tumors who recurred within 3 years) were detected by image analysis that would have been missed via grade alone.

### Time-to-event analysis

**Fig. 1:**
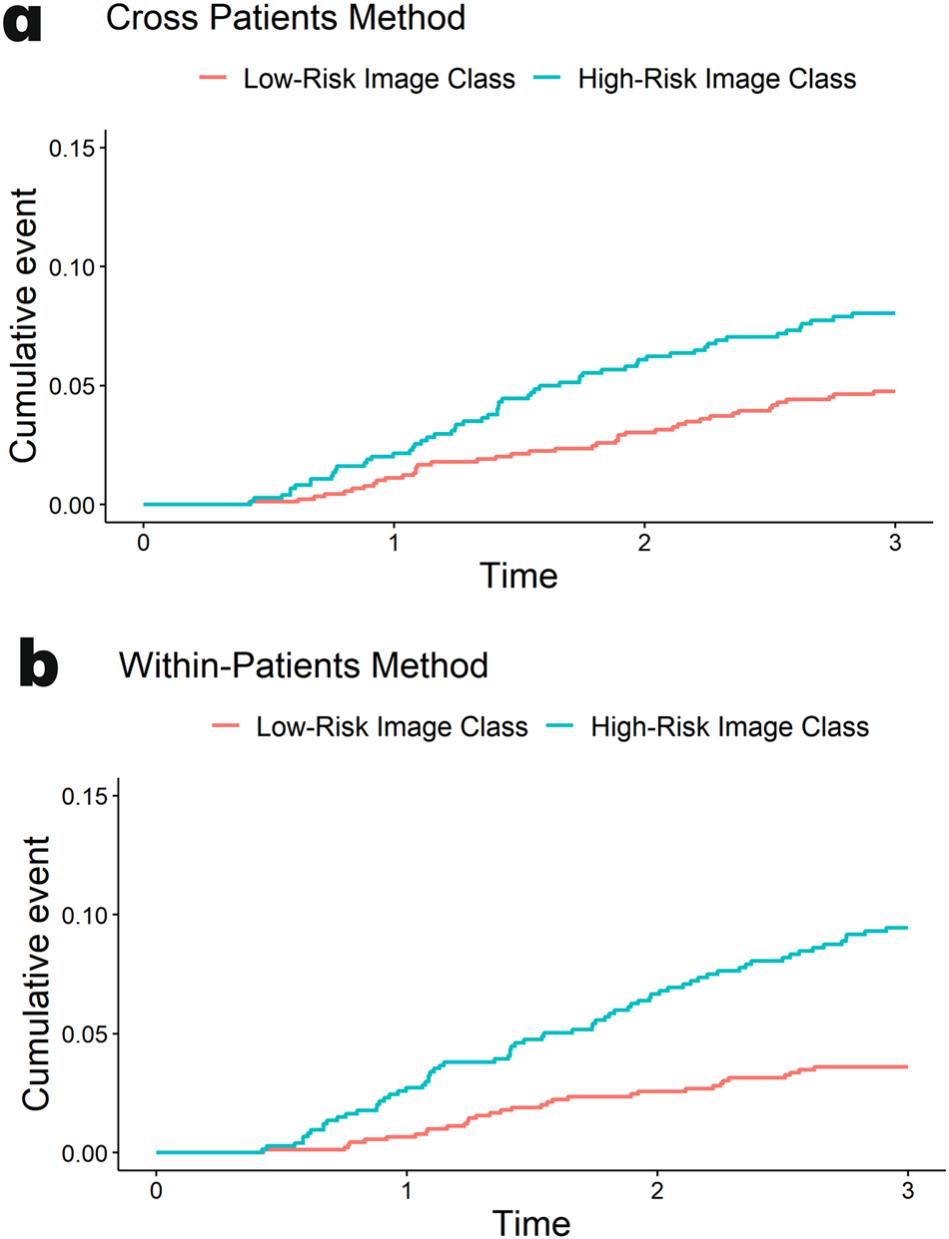
Kaplan-Meier plots for the cumulative incidence of recurrence. These plots were generated using (**a**) the cross-patients validation method and (**b**) the within-patients validation method. Cox proportional HR (95% CI) for cross-patients method: 1.73 (1.16, 2.57); HR (95% CI) for within-patients method: 2.70 (1.78, 4.11).

To also consider time-to-event (and not just binarized early recurrence vs. not), we evaluated both the within- and cross-patients predictors in time-to-recurrence based on Kaplan–Meier analysis (Fig. 1). Image-based classes from the within-patients approach (HR 2.70; 95% CI: 1.78, 4.11) had a slightly stronger hazard of recurrence than the cross-validation-derived classes (HR 1.73; 95% CI: 1.16, 2.57), but both were significantly associated with time to recurrence.

### Comparison with genomic assays

When comparing image-based classes to existing molecular metrics that represent risk of recurrence, specifically research-based versions of PAM50-derived ROR-PT and OncotypeDX scores, we observed that the image-based “high-risk” class was substantially enriched for individuals whose tumors had also been classified as high-risk by either ROR-PT or OncotypeDX (Table 3). The cross-patients approach resulted in an image-based high-risk class with the highest proportion of molecularly high-risk individuals [OncotypeDX RFD (95% CI): 15.0% (8.6, 21.3), ROR-PT RFD (95% CI): 21.5% (14.2, 28.5)], though the high-risk class resulting from the within-patients approach was still substantially associated with high-risk molecular features [OncotypeDX RFD (95% CI): 11.7% (5.3, 18.1), ROR-PT RFD (95% CI): 17.4% (10.0, 24.5)].

**Table 3:** Relative frequency differences (RFDs) for RNA-based risk of recurrence classifiers.

	Image-based Low Risk *N* (%)	Image-based High Risk *N* (%)	RFD (95% CI)
Cross-Patients Method			
OncotypeDX Low-Intermediate	290 (58.5)	206 (41.5)	Ref
OncotypeDX High	197 (44.7)	244 (55.3)	13.8% (7.4, 20.1)
ROR-PT Low-Intermediate	524 (56.2)	408 (43.8)	Ref
ROR-PT High	74 (34.7)	139 (65.3)	21.5% (14.2, 28.5)
Within-Patients Method			
OncotypeDX Low-Intermediate	280 (56.5)	216 (43.5)	Ref
OncotypeDX High	199 (45.1)	242 (54.9)	11.3% (4.9, 17.7)
ROR-PT Low-Intermediate	525 (56.3)	407 (43.7)	Ref
ROR-PT High	83 (39.0)	130 (61.0)	17.4% (10.0, 24.5)

Our image-based high-risk groups were significantly enriched for individuals who were categorized as high-risk based on RNA-based risk of recurrence classifiers.

## Discussion

We applied convolutional neural networks to detect early recurrence in the diverse, clinically well-annotated Carolina Breast Cancer Study. We found that image-based features predict survivorship with accuracy, sensitivity, and specificity that are comparable to those for standard clinical markers such as estrogen receptor status and grade. The performance characteristics of image-based classifiers differed within strata defined by grade, suggesting that future optimization should consider training separate within strata of grade. However, these image-based classifiers predicted recurrence with significant hazard ratios, and showed associations with risk-based genomic signatures. Since genomic signatures were not used in training, the association with genomic data suggests promise for rapid, low-cost pre-screening of tumors that may need further genomic testing. The importance of image-based pre-screening may also be of increasing importance as the proportion of neoadjuvant-treated breast cancer cases increases^25^, because in neoadjuvant cases tissue for diagnostic purposes is limited to biopsy materials. It may also be advantageous that the image-based methods use the same data collected for diagnosis and do not require any additional laboratory steps.

Our analysis is unique in that we trained on recurrence rather than other genomic or clinical data. Previous machine learning studies have predicted breast cancer recurrence and survival, most commonly using clinical and demographic data as inputs to ML algorithms^26–29^. Lou et al. compared an array of computational methods on a registry data consisting of 1140 patients, using collected medical records as inputs to the machine learned classifier^26^. Our approach used a much smaller image dataset for training but does not assume that the clinical data are mediators of the recurrence outcomes, allowing us to discover features that may not be captured in other clinical data. The promising results obtained with a small sample size suggest that future, larger studies with more images may improve accuracy further.

Our recurrence-trained classifier also recapitulated genomic risk subtypes, with high image-based risk groups being more likely to have high genomic ROR-PT and OncotypeDX scores. Other researchers have predicted genomic scores from images. Whitney et al. used 178 breast tumor H&E images to predict RNA-based OncotypeDX scores^13^. Accuracy in that study was 74% for low-intermediate vs high OncotypeDX score. We did not compare to Oncotype DX, which limits our ability to directly compare our accuracy for a PAM50-based risk of recurrence score, but our results suggest that training on recurrence rather than recurrence score is a viable strategy. Also, within the CBCS, our group demonstrated that ML methods could be utilized to predict tumor features such as ER status, grade, and subtype using images and data from CBCS3^12^. Accuracy in that analysis for prediction of high vs low-medium ROR-PT score was 75%, which is slightly higher than our results for recurrence. However, the training set for that analysis was much larger than the recurrence vs. non-recurrence dataset used here and the outcome was more common. Thus, future analyses with larger datasets should evaluate the optimal method for identifying high risk specimens.

Application of breast tumor tissue core images to predict the binary recurrence outcomes is a difficult problem because of a few key challenges. First, the input data from CNN (512 times the number of samples) is much more high-dimensional than researcher-selected features like grade, stage, and other clinical characteristics. Second, early recurrence rates in breast cancer are fortunately relatively low, but this results in recurrence data from breast cancer cohorts being highly imbalanced. Using a matching scheme to match each recurrent case with a non-recurrent participant allowed us to overcome some of the challenges of using machine learning techniques while working with such a strongly imbalanced data set.

Higher sensitivity when training within-patients suggests some similarity of the images in training was being leveraged in testing and raises the intriguing hypothesis that repeated samples of images from patients have some individuality or ‘identifiability’. Whether this identifiability is clinically meaningful merits further investigation. On the molecular level, Perou et al. suggested that tumors are individuals and that this individuality may be targetable for precision medicine^30^; if tumors are similarly individual on a histological level, perhaps machine-learning techniques to evaluate histologic distinctions across a tumor could be used to identify subgroups or to study tumor evolution between biopsies and excisions/mastectomies. In any case, establishing the reproducibility of classification across samples from a given tumor is an important future direction if histologic biomarkers are to be used for risk prediction.

In summary, our proposed image-based approaches achieve competitive prediction accuracies on the order of established biological and clinical markers (i.e., grade and ER status), with balanced sensitivity and specificity. Among patients in the low/intermediate grade subgroup, both approaches were more sensitive than ER status. This analysis underscores the promise of training histopathologic predictors directly on recurrence rather than clinical surrogates, and emphasizes the need for larger, collaborative analysis of breast cancer outcome where sufficient event sizes and inter-study comparisons can be made. The benefits of a histologic approach for risk stratification could be significant, particularly for low-grade patients where the current markers (grade and ER) are not sensitively capturing risk of recurrence.

## Methods

### Study population

CBCS3 is a prospective, population-based cohort of 2998 women with incident invasive breast cancer recruited from 44 counties in North Carolina between 2008 and 2013. First, primary breast cancer cases were identified using rapid case ascertainment in collaboration with the North Carolina Central Cancer Registry. Eligible women were between 20 and 74 years old. Black and young (<50 years old) women were oversampled to each represent 50% of the population. The study was approved by the University of North Carolina Institutional Review Board in accordance with U.S. Common Rule. All study participants provided written informed consent prior to study entry. This study complied with all relevant ethical regulations, including the Declaration of Helsinki.

Breast cancer recurrence was ascertained by patient self-report at annual telephone follow-ups and then confirmed by medical record. Formalin-fixed, paraffin-embedded (FFPE) tumor blocks were obtained from participating medical centers for participants with available tissue. Tumor blocks were obtained for 1743 of the women enrolled in the study and were reviewed by the study pathologist (JG). From tumor-enriched regions selected by the pathologist, between one and four 1-mm tumor cores were sampled and embedded in tissue microarrays (TMAs) at the Translational Pathology Laboratory at UNC-Chapel Hill. TMA slides were sectioned (5-μm thickness) and top and bottom sections were stained with hematoxylin and eosin (H&E) and scanned at 20x magnification. In the balanced dataset, the 202 participants corresponded with 704 H&E core images of approximately 3000 × 3000 pixels and each participant had between two and four 1-mm tumor cores.

Tumor grade was determined centrally by the study pathologist, except where whole slide images were unavailable for secondary review and the originally reported (“clinical”) tumor grade was used (*n* = 7) or where both slides and clinical grade were missing (*n* = 39). From 1743 women with TMA images, participants were excluded if missing grade or if tissue was insufficient for VGG16 CNN (i.e., core damaged or section folded, tumor was depleted, *n* = 30). Because this study was aimed at evaluating recurrence, women with metastatic disease at diagnosis were excluded (*n* = 40), resulting in a final eligible population of 1644 women, corresponding to a total of 5969 core images. Approximately 7% of the study population experienced an early recurrence (*n* = 101), defined as recurrence within three years of diagnosis. To construct a balanced dataset of recurrent and non-recurrent cases, we matched recurrent cases to non-recurrent participants 1:1 on age (defined in 5-year bins).

### Gene expression assays

Where available, additional FFPE specimens from CBCS3 participants were obtained for RNA extraction. RNA was isolated using RNeasy FFPE Kits (Qiagen) and Nanostring gene expression assays were performed at UNC Chapel Hill in the Translational Genomics Lab. Gene expression data were cleaned and normalized as described previously^31^. Of the 1543 women included in the study, 1145 women had data on genes required for the PAM50 predictor, a research-only version of the Prosigna clinical assay. These genes were used to calculate a PAM50 risk-of-recurrence (ROR) score. For this study, the ROR-PT score was used, which additionally incorporates information on the PAM50 subtype, proliferation score (P), and tumor size (T)^7^. These scores were then categorized into low-intermediate and high risk. Data on the 21 genes included in the OncotypeDX score assay were available for 937 of the 1543 women on study. These genes were used to approximate OncotypeDX scores for these patients, which were then categorized into low-intermediate (<26) and high (26+) risk.

### Model specification and validation

#### Image preprocessing and feature extraction

The appearance of core images was standardized in each color channel to have mean equal to zero and standard deviation equal to one. We used a CNN^32^ to extract feature representations of core images (*n* = 704 cores from 202 participants). The CNN first applies convolutional filters followed by pooling operations. Lower-level layers learn generic image features such as edges and shapes, intermediate level layers capture increasingly complex properties like shape and texture, and higher-level layers learn global concepts that describe the semantic meaning of the images^32,33^. The parameters of such a network are the weights of the convolutional filters and are learned from the data in an adaptive manner, creating a hierarchically set of features of increasing abstraction.

We used the VGG16^23^ network that was pre-trained on the ImageNet dataset, without alteration. We explored using other networks (e.g., Resnet), but VGG16 resulted in better cross-validation and test set accuracy. ImageNet^34^ contains 1.2 million images, all of which belong to one of 1000 ImageNet object categories^35^, and although the extant categories are different from histology images, the pre-trained weights transfer well to feature extraction from tissue sections. To use the pre-trained VGG16 network to extract features of the core images, we feed the core images as input into the VGG16 network. Each layer transforms the features obtained from the previous layer based on its parameters and learns concepts like shape and texture that are complex enough for generalization but not too specific to images in ImageNet as to be inapplicable to histology images. In principle, one can use the features at any layer as the feature representation of the input image. Similar to the approach used by Couture^12^, we evaluated performance of the features extracted from different layers of the pre-trained VGG16 network. We ran a grid search over the feature extraction layers on 90% of the data (training set) and evaluated the performance on the remaining 10% (validation set). The 7th layer had highest validation accuracy and was therefore selected for model development. A total of 704 cores across 202 participants were used to extract a 512 × 64 × 64-dimensional matrix of image features. Spatial mean pooling was used to calculate to produce a feature vector of length 512 for each tumor core for use in prediction analysis.

We first attempted to use fully connected layers, but this resulted in overfitting. Therefore, we used a support vector machine (SVM) to classify the patient-level features that predict binary recurrence. An SVM^24^ is a classification algorithm that finds a linear decision boundary to separate the two classes (here, recurrent vs non-recurrent). The foundational idea of SVM is that if one interprets the margin between a data point and the decision boundary as the difficulty of classifying that data point (i.e., the smaller the margin is, the more difficult it is to classify that data point), we seek an optimal decision boundary that maximizes the margin, thereby minimizing the classification difficulty. After locating the decision boundary, an SVM predicts the class assignments of the data points based on which side of the decision boundary those points are on. We used those predicted class assignments for further analysis of the recurrence prediction.

We also explored training a neural classifier for predicting early recurrence in an end-to-end fashion; however, due to the small sample size of our balanced data set, the trained deep network overfit the training data and poorly distinguished recurrences in the test set.

#### Validation datasets

**Fig. 2:**
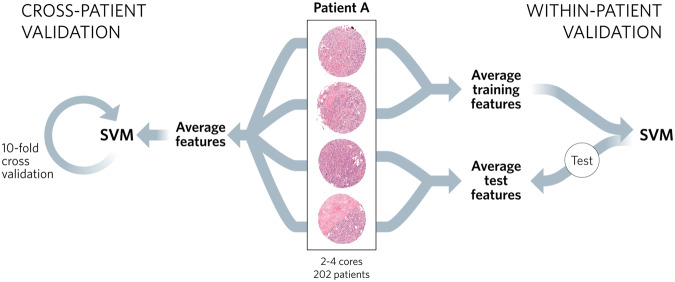
Workflow for cross-patients validation (left) and within-patients validation (right) approaches. For the cross-patients validation, image features from all of each patient’s cores were extracted using VGG-16 and averaged as a feature representation for the patient. An SVM was trained on the features of the cohort and tested using 10-fold cross-validation. For the within-patients validation, image features from half of each patient’s cores were extracted using VGG-16 and averaged as the feature representation for each patient. An SVM was trained on the features of this first half of the images. Image features from the second half of the patient’s cores were then averaged and used to test this classification.

Ideally, data is split into training, validation, and test sets to evaluate the accuracy of ML algorithms; however, the balanced data set (*n* = 202) was small, and therefore, we assessed performance via two methods. First, our goal was to have a single feature vector to represent each patient. To this end, we performed a *cross-patient validation* (Fig. 2), in which we averaged the feature vectors of multiple core images per patient to create a single patient-specific feature vector. We then performed ten-fold cross-validation to train and evaluate the model for predicting recurrence, with one-tenth held back for testing in each iteration. We confirmed that as we added additional folds for k-fold validation (i.e., as we increased k), we increased our accuracy from 56% at 2-fold to 62% at 10-fold. Second, we performed a *within-patient validation* (Fig. 2). In this method, we took advantage of multiple tumor cores for each patient and trained the model using the feature vectors on half of each patients’ cores, testing the model on the second half. This latter method assumes that the core images belonging to each patient are independent, which is unlikely given that the correlation between image features within a patient (average cosine similarity within individual patients’ image features = 0.91) was higher than correlations between patients (average cosine similarity between patients’ image features = 0.84). However, we were interested in estimating how ‘individuality’ of a given tumor contributes to predictive accuracy, and thus, we considered within-patient methods as an optimistic estimation of model performance and compared the results to those obtained by cross-validation.

### Statistical analysis

The predictive value of image-based classes was assessed using sensitivity, specificity, and overall accuracy of prediction. 95% confidence intervals were produced for these measures using the normal approximation of a binomial proportion. Following development of a binary classification scheme, we performed time-to-event analyses to assess relationships between the SVM-derived image classes and recurrence within the full cohort. Cox proportional hazards models were used to estimate hazard ratios and 95% confidence intervals. Relationships between image-based classes and recurrence were visualized with Kaplan–Meier curves. Generalized linear models with identity link and binomial family were used to estimate relative frequency differences (RFDs) and 95% confidence intervals to describe associations between image classes and molecular risk scores (OncotypeDX and ROR-PT).

## Data Availability

The data analyzed in this study are available from the Carolina Breast Cancer Study (https://unclineberger.org/cbcs/). Restrictions apply to the availability of these data, which were used under data use agreements for this study. Data is not publicly available; however, investigators may submit a letter of intent to gain access upon reasonable request.
